# Fine Mapping of the QTL *qRLP12* That Controls Root Length Under Polyethylene glycol-Induced Drought Stress During the Early Seedling Stage of Sesame

**DOI:** 10.3390/ijms26072886

**Published:** 2025-03-22

**Authors:** Junchao Liang, Yanxin Deng, Xiaowen Yan, Zhiqi Wang, Pan Zeng, Meiwang Le, Hongying Zhou, Jian Sun

**Affiliations:** 1 Crop Research Institute, Jiangxi Academy of Agricultural Sciences, Jiangxi Province Key Laboratory for Genetic Improvement of Oilcrops, Nanchang 330200, China; njljc@163.com (J.L.); dengyanxin0717.stu@yangtzeu.edu.cn (Y.D.); yanxiaowen1983@126.com (X.Y.); 18679145645@126.com (Z.W.); 15271750715@163.com (P.Z.); mwyuecarl@163.com (M.L.); zhouhongying99@163.com (H.Z.); 2 College of Agriculture, Yangtze University, Jingzhou 434025, China

**Keywords:** sesame, root length, *qRLP12*, drought stress, fine mapping

## Abstract

A deeper root system can improve the efficiency of water and nutrient absorption from soil; therefore, genetic improvements to the root length of crops are essential for yield stability under drought stress. We previously identified a stable quantitative trait locus (QTL) *qRLP12* for root length under polyethylene glycol (PEG)-induced drought stress in a Jinhuangma (JHM, sensitive)/Zhushanbai (ZSB, tolerant) recombinant inbred line (RIL) population. To validate and fine map this QTL, in this study, a secondary F_2_ population was constructed, and the genetic effect of the target QTL was validated by comparing the phenotype data of different genotypes. Using newly developed markers, 14 genotypes of recombinant F_2_ individuals were obtained. A phenotypic analysis of homozygous recombinant progeny lines narrowed *qRLP12* to a 91 kb region. Seven putative predicted genes were identified in the target region, among which *LOC105165547*, a callose synthase gene, was the only one containing nonsynonymous variations in the coding region between two parents. Quantitative real-time PCR analysis revealed that *LOC105165547* was significantly induced by PEG stress in the *qRLP12*+ line. These indicated that *LOC105165547* might be the candidate gene for *qRLP12*, which is responsible for root length subjected to PEG stress. Our results provide a favored gene resource for improving root length under drought stress in sesame.

## 1. Introduction

Drought is recognized as one of the most challenging abiotic stresses affecting crop productivity worldwide. The percentage of the planet affected by drought has more than doubled in the last 40 years, and during the same period, droughts have impacted more people worldwide than any other natural hazard [1]. This alarming trend underscores the urgent need for effective strategies to enhance drought tolerance in crops. Deep root systems in terrestrial crops allow them to avoid drought stress by absorbing water from subsoil. Improving the root length under water shortage conditions is therefore a critical strategy to enhance drought tolerance in crops [2]. Research has shown that deeper rooting can enhance grain yield under drought conditions in crops such as maize [3], sorghum [4], wheat [5], and rice [6]. Therefore, understanding the genetic and molecular mechanisms underlying root development is essential for improving agricultural sustainability and addressing food security challenges.

Sesame (*Sesamum indicum* L., 2n = 26) is one of the world’s most important oilseed crops, often referred to as the “queen of oilseeds” due to its rich nutrient profile, including tocopherols, lignans, proteins, and plenty of various mineral elements [7,8]. Although sesame is considered a typical “survivor crop” since it has relatively strong tolerance to water deficits, it is particularly vulnerable to drought stress at the germination and flowering stages, primarily due to its shallow root characterization [9,10]. Moreover, the increasing frequency and intensity of drought events, exacerbated by climate change, have further heightened the risk to sesame production over recent decades [11,12]. Major sesame-producing areas worldwide, such as West and Central Africa [13], India [14], and China, frequently experience drought stress during sesame growth season. In China, flash droughts and seasonal droughts frequently coincide with the sesame sowing period in most parts of the main sesame-producing areas, especially in southern China; this significantly hampers sesame seedling growth and production potential [11,15,16]. This highlights the pressing need to enhance the drought tolerance of sesame to ensure yield stability.

Despite the existing knowledge surrounding drought tolerance, significant gaps remain in our understanding of the genetic mechanisms regulating root growth under drought stress in sesame. So far, several studies using transcriptome analysis have revealed multiple transcription factors (TFs), such as ERF [17], MYB [18], bZIP [19], NAC [20], and HD-ZIP [21], participating in the response to drought stress in sesame. Some of these TFs, including *SiERF5* [22], *SiNAC104* [22], *SiMYB75* [23], and *SiMYB77* [24], have been found to improve root elongation and enhance tolerance to drought stress in transgenic *Arabidopsis thaliana* and tobacco plants. Besides these, some other genes, such as the osmotin-like proteins gene (*SindOLP*) and S-adenosylmethionine synthetase gene (*SiSAM*), regulate drought tolerance through various pathways [25,26]. Overexpression of *SindOLP* in sesame could provide longer roots and enhanced tolerance against drought, salinity, and the charcoal rot pathogen [25], while *SiSAM* was found to be related to capsule number and stem length under drought stress in a genome-wide association (GWAS) study examining drought tolerance-related traits at the flowering stage [26]. The latter gene may improve drought tolerance by modulating the polyamine level and ROS homeostasis [26]. Another sesame orphan gene, ‘*Big Root biomass*’, improved yield parameters under normal growth conditions and increased drought stress sensitivity when overexpressed in *Arabidopsis thaliana* plants [27]. In addition, transcriptome profiling of root and leaf samples has revealed hundreds of genes and microRNAs participating in drought response in sesame [28,29,30,31,32]. However, while some of these identified TFs or genes were found to be able to improve root growth under drought stress in transgenic *Arabidopsis thaliana* and tobacco experiments, major QTLs or candidate genes for root growth under drought stress remain largely unexplored in sesame. To our knowledge, only three stable major QTLs for root length under drought stress were identified by far, which was performed by our lab [33]. The genetic mechanism and underlying functional genes that govern root growth under drought stress in sesame are still unknown, representing a critical knowledge gap in the field. Validation, fine mapping, and cloning of major QTLs are essential for understanding their function and applying them in sesame improvement.

We previously identified a major QTL for the main root length under polyethylene glycol (PEG)-induced drought stress (RLP) at the early seedling stage using an RIL population derived from Jinhuangma (JHM, sensitive) and Zhushanbai (ZSB, tolerant) [33]. This QTL explained 11.85–14.46% of the root length under PEG stress variations across different experiments, with the landrace ZSB conferring the favorable allele for longer roots [33]. Here, the drought-tolerant line RA11 and drought-sensitive line RA171 selected from the JHM/ZSB RIL population were used to construct a large F_2_ mapping population to fine map *qRLP12*, develop robust markers for breeding, and identify the underlying gene for root length under drought stress in sesame.

## 2. Results

### 2.1. Validation of qRLP12 for Root Length Under PEG Stress

**Figure 1:**
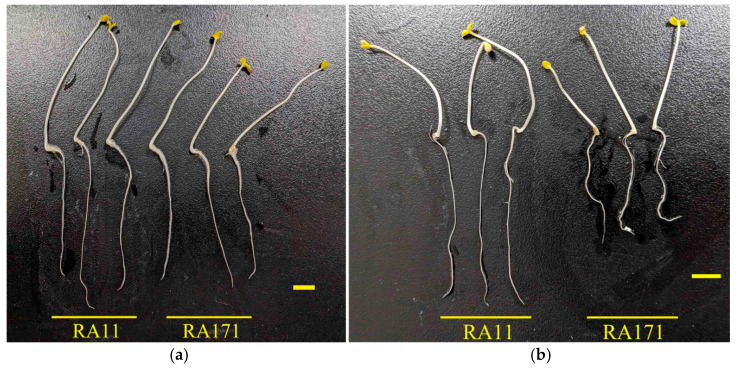
Root phenotypes of the mapping parents RA11 and RA171. (**a**): water condition; (**b**): 15% PEG treatment. Scale bars represent 1 cm.

**Figure 2:**
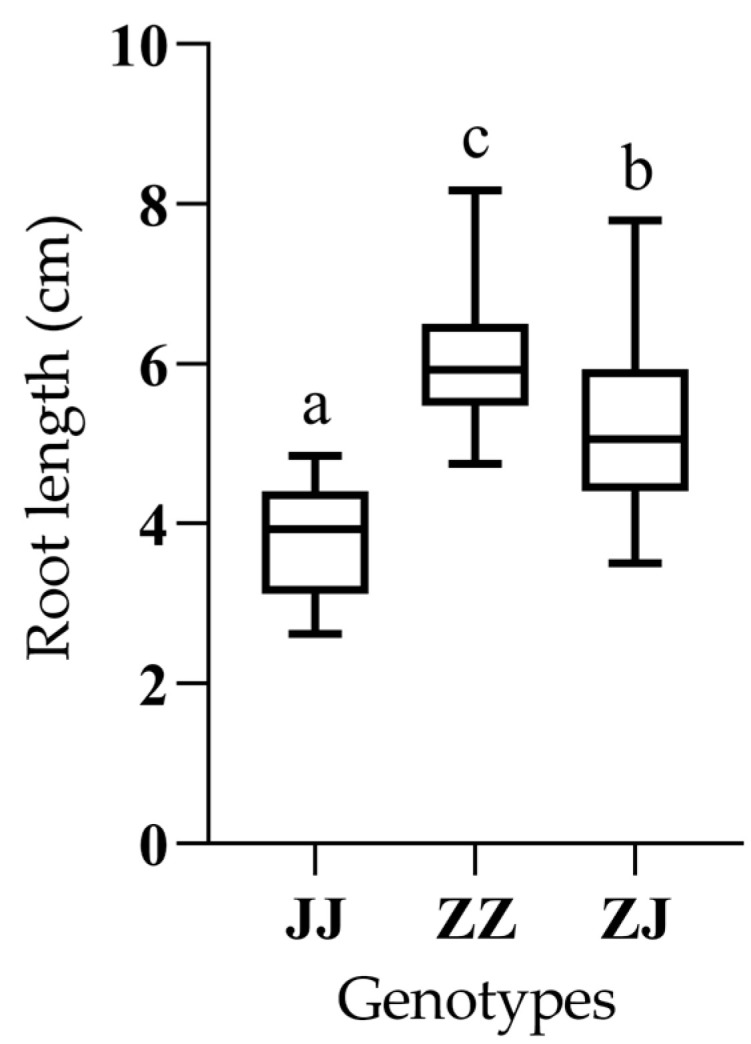
Root length under PEG-stress of F_2:3_ lines grouped according to their genotypes of flanking markers *Z9* and *Z10*. ZZ: homozygous ZSB genotype; JJ: homozygous JHM genotype; ZJ: heterozygous genotype. Different letters above indicate significant differences at *p* = 0.01 between genotypes.

*qRLP12* was initially mapped to a 4.2 cM genetic interval in the RIL population derived from JHM and ZSB [33]. To further verify the genetic effect of *qRLP12*, we constructed a secondary F_2_ population by using two RILs: RA11 and RA171. RA11 harbored only one QTL (*qRLP12*) for root length under PEG stress, while RA171 contained no such QTL. All the F_2_ plants were planted in nylon net houses to obtain F_2:3_ lines. Under PEG stress, RA11 had a significantly longer root length than RA171, while the root length under water control conditions showed no difference between these two lines (Figure 1, Table S1). Two flanking markers, *Z9* and *Z10*, were developed based on variations from our previous whole-genome resequencing data. After genotyping with *Z9* and *Z10*, one hundred and six F_2:3_ lines showing no cross-over between these two markers were used for phenotyping. According to the marker genotypes of *Z9* and *Z10*, the F_2:3_ lines could be divided into three groups, including Jinhuangma type (JJ), Zhushanbai type (ZZ), and heterozygous type (ZJ). Analysis of variance components for the phenotypic data revealed that the RLP mean of the ZZ group was significantly larger than that of the JJ group (*p* < 0.01) (Figure 2, Table 1 and Table S2). No significant variation was detected among different replicates (Table S3). The distribution of RLP in the heterozygous group overlapped with those of both the JJ and the ZZ groups. These results suggest that *qRLP12* was segregated in this F_2_ population with the enhancing allele derived from ZSB. Therefore, *qRLP12* was identified as a stable locus and suitable for further fine mapping and cloning.

### 2.2. Fine Mapping of qRLP12

**Figure 3:**
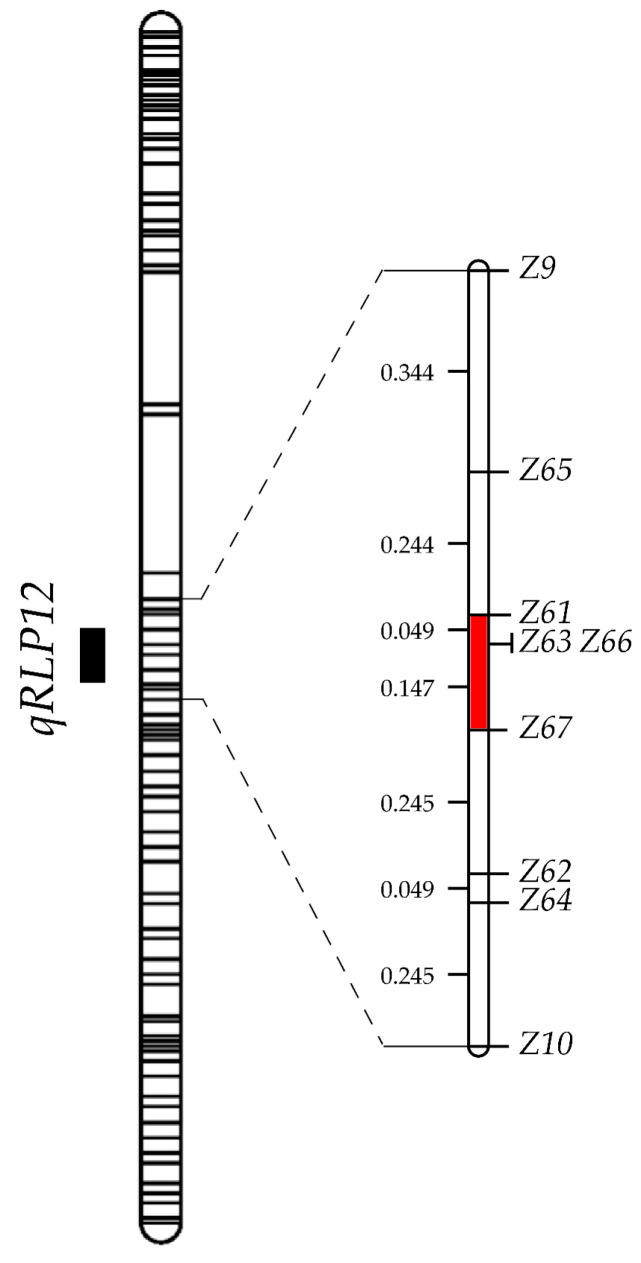
High-resolution linkage map of the *qRLP12* interval. The QTL interval shown to the left of the map was excerpted from Liang et al. (2021) [33]. The red rectangle shown in the map represents the co-segregation region of *qRLP12*.

To refine the position of *qRLP12*, we screened 1025 F_2_ plants derived from RA11 and RA171 for recombinants between markers *Z9* and *Z10*. This led to the identification of 27 plants with cross-over events between these two markers. To saturate this target region, seven additional markers, *Z65*, *Z61*, *Z66*, *Z63*, *Z67*, *Z62*, and *Z64*, were developed based on variations between JHM and ZSB according to previous resequencing data (Table S4, Figure 2). Among the 27 recombinants, we identified 14 genotypes by surveying with these markers in the target interval. No cross-over event was observed between the two markers *Z66* and *Z63* in our mapping population. By means of linkage analysis using the genotype data of recombinants, a high-resolution map was constructed (Figure 3). These markers were mapped to a 1.32 cM interval. Homozygous F_3_ progenies of recombinants were singled out by using these markers and planted in a net house to obtain two groups of homozygous F_3:4_ lines (including recombinant and non-recombinant types) for phenotyping.

**Figure 4:**
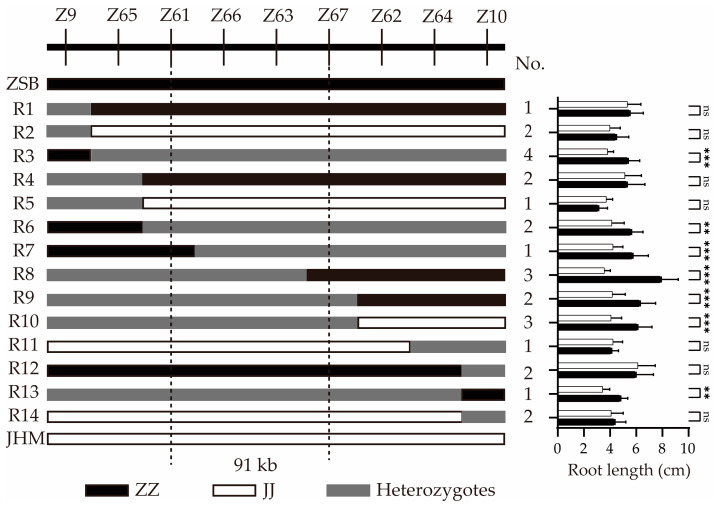
Genotypes (**left panel**) and the corresponding phenotypic assays (**right panel**) of *qRLP12* recombinants. Dark, open, and gray rectangles represent the ZSB genotypes, JHM genotypes, and heterozygotes, respectively. The vertical dashed lines define the interval harboring *qRLP12*. Statistical analyses of phenotypes between different genotypes from self-pollinated progenies of each recombinant are shown with **, ***, and ns, significant at *p*  <  0.01 and *p*  <  0.001, and non-significant, respectively.

After comparisons and a two-way ANOVA of the phenotype data among different homozygous progeny F_3:4_ lines of recombinants, significant differences (*p* < 0.01) in RLP were detected only between different groups of progeny lines of seven recombinants (R3, R6, R7, R8, R9, R10, and R13) that had heterozygous regions delimited by the markers *Z61* and *Z67*. The effect of different replications on phenotype was not significant (Table S5). Among these progenies, the lines harboring homozygous ZSB alleles in the *Z61*–*Z67* interval exhibited longer RLP. On the other hand, the remaining recombinant genotypes (R1, R2, R4, R5, R11, and R14) carried homozygous segments in the same interval, and no significant differences in RLP were detected between their progeny groups (Figure 4). Consequently, *qRLP12* was finally mapped to a 91 kb region between markers *Z61* and *Z67* based on the location of critical recombination events (Figure 4).

### 2.3. Candidate Genes in qRLP12 Region

By using the sesame reference genome v1.0 database, a total of seven predicted genes were identified in the targeted 91 kb [34] (https://www.ncbi.nlm.nih.gov/datasets/genome/GCF_000512975.1/, accessed on 5 March 2024). These genes were annotated to encode callose synthase 7 (*LOC105165547*), conserved oligomeric Golgi complex subunit 5 (*LOC105165546*), GDSL esterase/lipase (*LOC105165545*), protein VACUOLELESS1 (*LOC105165544*), 3-hydroxyisobutyryl-CoA hydrolase-like protein 5 (*LOC105165543*), heavy metal-associated isoprenylated plant protein (*LOC105165542*) and subtilisin-like protease (*LOC105165541*). Based on the previous whole-genome resequencing data for the two parents, we discovered 14 SNPs and four insertions and deletions (InDels) in the 91 kb candidate region. Among these seven genes, only the callose synthase 7 (*LOC105165547*) contained nonsynonymous variations between the two parents. The subtilisin-like protease gene (*LOC105165541*) harbored one SNP in its fifth intron. The flanking marker Z67 was developed based on the corresponding SNP in *LOC105165541*, while *LOC105165547* had 10 variations, including two InDels and eight SNPs (Tables S6 and S7). Two nonsynonymous substitutions (A to G and T to C) in *LOC105165547* resulted in amino acid changes (Thr to Ala and Val to Ala) between ZSB and JHM. Two insertions of 35 bp and 37 bp were detected in the last exon of *LOC105165547* in JHM, leading to a premature stop codon (Table 2, Figure S1). The insertion sites of these two fragments were only 5 bp apart. Notably, the cleaved amplified polymorphic sequences (CAPS) marker *Z66* and InDel marker *Z63* in the *qRLP12* co-segregation region were designed based on the missense SNP (A to G) and the two insertions in *LOC105165547*. These suggested that the callose synthase-encoding *LOC105165547* was the most likely causal locus for *qRLP12*.

### 2.4. Expression Analysis of Candidate Genes

**Figure 5:**
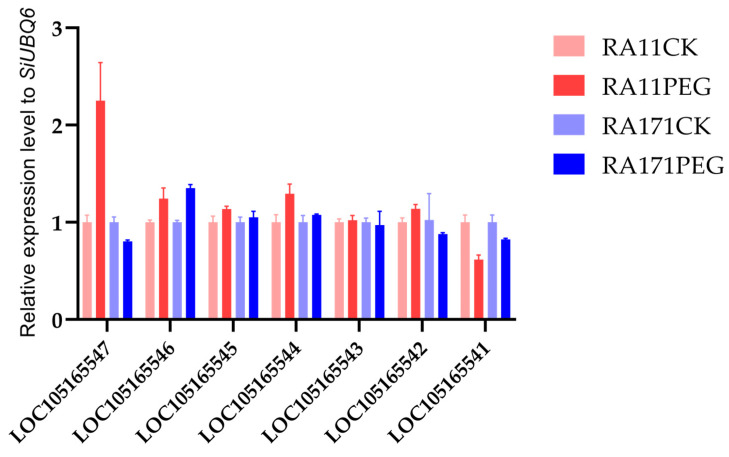
Expression levels of the seven candidate genes in the roots of RA11 (*qRLP12*+) and RA171 (*qRLP12*-) subjected to PEG-stress or water control. *SiUBQ6* was used as an internal control.

To further identify the candidate gene for *qRLP12*, quantitative real-time PCR (qRT-PCR) was used to detect the expression levels of all seven predicted genes of the target region in RA11 and RA171 subjected to PEG stress (Figure 5). Comparison with water control treatment, *LOC105165546* was significantly induced in the roots of both RA11 and RA171 under PEG stress by similar degrees (*p* < 0.01). Two genes, *LOC105165544* and *LOC105165547*, were significantly up-regulated only in RA11 (*p* < 0.01). *LOC105165541* was significantly down-regulated in both RA11 and RA171 (*p* < 0.01 for RA11, *p* < 0.05 for RA171). The expression level of *LOC105165547* was significantly suppressed in RA171 (*p* < 0.05). There was no difference in the expression of the other three genes (*LOC105165545*, *LOC105165543*, and *LOC 105165542*) between PEG stress and water conditions in the root of both RA11 and RA171. These results further supported our conclusion that *LOC105165547* might be a good candidate gene for PEG-induced drought stress tolerance at the *qRLP12* locus.

## 3. Discussion

Deep roots are often favored for plant seedling establishment and growth, especially during periods of water scarcity [35,36,37,38]. As roots are the hidden part of plants, their traits are relatively more difficult to investigate than those of above-ground parts. To date, few genes are associated with root development and root-related traits for drought tolerance in sesame. Phenotypic investigations using hydroponic systems and field experiments have revealed that sesame roots have great morphological and anatomical diversity [39,40]. This allows the identification of major loci/genes for root traits through genetic analysis approaches, such as linkage mapping and GWAS. By using GWAS, an orphan gene, *BRB*, associated with root number and root dry weight, was identified on LG15 [27]. *BRB*-overexpressing *Arabidopsis* lines displayed reduced total lateral root length and fresh root weight [27]. Besides this gene, six QTLs associated with root length in hydroponic growth conditions were also detected on LG1, LG4, LG5, LG7, and LG10 using the same diversity panel [27]. However, no major genes for root traits under drought stress were cloned in sesame so far, and the genetic mechanism of root growth under water deficits in sesame remained unclear.

In our previous study, we identified three stable QTLs associated with root length under PEG stress in a JHM × ZSB recombinant inbred lines population [33]. Among them, *qRLP12* located on LG6 had the highest LOD value and strongest effect, meaning that it could be an important gene for better drought tolerance at the early seedling stage in sesame. The ZSB allele of *qRLP12* conferred a longer root length. Here in this study, using 27 recombinants from the secondary F_2_ population, we fine mapped the *qRLP12* locus to an interval between the markers *Z61* and *Z67*, spanning a 91 kb region on LG6 according to the sesame reference genome sequence v1.0. To our knowledge, this is the first fine map of a root length QTL under drought conditions in sesame. Our results provide the first understanding of the genetic control of root length response to drought stress in sesame. The genome location of the *qRLP12* interval is different from that of any other reported root trait QTL or drought tolerance-related QTL in sesame [26,27,41], suggesting a potential unique genetic pathway influencing root development and drought response in sesame. Further studies on the interaction of *qRLP12* with other drought tolerance QTLs may reveal the genetic mechanism of water deprivation response in sesame.

There are seven annotated genes in the target region of *qRLP12* according to the sesame reference genome database v1.0. Three of them have been highlighted in association with root development or growth. In particular, *LOC105165545* encodes GDSL esterase/lipase (GELP), which have been linked to root development through hormone-mediated signaling pathways in rice [42], soybean [43], and *Arabidopsis* [44]. *LOC105165541* encodes subtilisin-like protease potentially involved in regulating root growth or drought tolerance [45,46]. However, our genomic resequencing data revealed no functional polymorphism in *LOC105165545* between the two parent lines used in this study. Although *LOC105165541* harbors one missense mutation, this SNP was converted into the flanking marker *Z67* and mapped at the boundary of the *qRLP12* interval (Figure 3 and Figure 4). The key recombinant, R8, carrying a homozygous ZSB type in the marker *Z67* locus, showed segregating phenotypes among the progeny lines (Figure 4). These results indicated that *LOC105165545* and *LOC105165541* could be excluded as the *qRLP12* candidate. The other gene, *LOC105165547* encoding callose synthase 7 gene (here designated as *SiCalS7*), contains two InDels and two nonsynonymous substitutions in its open reading frame (Tables S6 and S7). The 35 bp insertion of *SiCalS7* in JHM caused a premature termination. Moreover, a gene expression analysis revealed that *SiCalS7* was significantly induced under PEG treatment in the *qRLP12*+ line RA11 but down- regulated in the *qRLP12-* line RA171 (Figure 5). A comparison of the 2 kb promoter region of *SiCalS7* revealed an SNP and a 2-bp InDel between two parents (Table S6), leading to a change in the CCAAT-box element, which is a typical stress-responsive element [47], indicating that these variations may be the cause of the differential expression of *SiCalS7*. Therefore, *SiCalS7* is strongly suggested as the candidate gene for *qPLP12*.

*SiCalS7* is homologous to *Arabidopsis CALLOSE SYNTHASE 7/GLUCAN SYNTHASE-LIKE7 (CALS7/GSL7*, *At1g06490)*, which belongs to glycosyltransferase family 48 (GT48) [48]. This enzyme is required for callose biosynthesis during phloem development [49]. It has been proposed that callose may serve as an induced defense mechanism in response to both biotic and abiotic stresses [50]. Callose deposition and trafficking in the cell wall play a crucial role in tissue development and responses to environmental stresses, while callose synthase genes regulate the level of callose in plants [51,52]. During root development, CALS7 is reported to be highly and specifically enriched in phloem sieve elements, which possess numerous symplastic connections to adjacent cells [53,54]. Loss-of-function mutations in *CALS7* result in complete loss of the callose lining of sieve plate pores between phloem sieve elements in roots [55]. Callose synthase genes can be activated by various biotic and abiotic stresses, and the accumulation of callose has been found to contribute to enhanced root cell walls and offer a physical barrier against drought stress [51]. For example, in soybean, callose accumulation in seedlings was a response to water deficit in roots [55]; in barley, PEG treatment could lead to drought-resistant genotypes accumulating higher levels of callose in their root systems compared to drought-sensitive genotypes [56], and high tolerance to drought and salt stress in Tibetan wild barley is closely related to enhanced callose [57]. Elevated callose content may impede the plant’s defense mechanisms against drought by enhancing its water retention capabilities, which could be associated with improved water use efficiency. Further mutagenesis studies or complementation experiments are needed to examine the function of *SiCalS7* and how it regulates root growth under drought stress. Recently, the clustered regularly interspaced short palindromic repeats (CRISPR)/CRISPR-associated protein 9 (Cas9) system had been successfully applied in sesame through hairy root transformation [58]. The biological functions of *SiCalS7* and its associated genomic variations could be confirmed using gene editing or high-efficiency overexpression transformation systems in future studies.

## 4. Materials and Methods

### 4.1. Plant Materials

RA11 is a recombinant inbred line (RIL) that carries *qRLP12*, and RA171 is a PEG stress-sensitive RIL containing no QTL for root length under drought stress. RA11 and RA171 were selected from an RIL population developed in our previous study from two parents, Jinghuangma (JHM) and Zhushanbai (ZSB), with contrasting drought tolerance [33]. An F_2_ population was developed by crossing the PEG-induced drought stress-resistant RIL RA11 and the susceptible RIL RA171 to fine map *qRLP12*. To verify the initial *qRLP12* mapping results, a total of 106 F_2:3_ lines were phenotyped and genotyped. All the plants were propagated in a nylon net house to prevent cross-pollination caused by insects.

### 4.2. Marker Development and Recombinant Screening

A total of 1025 F_2_ plants from the crossing of RA11 and RA171 were screened to identify recombinants in the *qRLP12* interval. Nine markers, including one SSR marker, four InDel (insertion and deletion) markers, one derived cleaved amplified polymorphic sequences (dCAPS) marker, and three CAPS markers, within the physical region delimiting *qRLP12* were developed based on a comparison of re-sequencing data for JHM and ZSB. All primers were designed with Primer Premier 6 (https://www.premierbiosoft.com/). The polymorphic primer pair sequences are shown in Table S4. Total genomic DNA was extracted from young, fresh leaves based on the CTAB method [59]. PCR was run on a Bio-Rad T100 thermal cycler (Bio-Rad Laboratories, Hercules, CA, USA) using the following protocol: 94 °C for 3 min, followed by 35 cycles of 94 °C for 30 s, 50–60 °C (depending on the specific primers) for 40 s, and 72 °C for 50 s, and a final extension at 72 °C for 5 min. PCR products were digested with corresponding restriction enzymes when needed, separated on agarose gel or 8% non-denaturing polyacrylamide gels (acrylamide/bis-acrylamide = 19:1 or 39:1) with 1 × TBE buffer, and visualized by means of silver staining. Recombinants were identified via genotyping with two flanking markers, *Z9* and *Z10*. The identified recombinants were then genotyped with seven additional markers to further differentiate recombinant event types. A linkage analysis was carried out with JoinMap^®^ v4 [60], and the genetic map was displayed with MapChart v2.32 [61]. The F_3:4_ lines of recombinants were also genotyped with newly developed markers to single out the homozygous ones for phenotyping. At least two homozygous lines for each genotype were used for phenotyping.

### 4.3. Phenotype Evaluation

The root length under polyethylene glycol (PEG)-simulated drought stress (RLP) was evaluated according to the methods used in our previous study [33]. Thirty-five mature seeds of each line were surface sterilized with 75% ethanol for 5 min, rinsed five times with sterile water, and dried at 25 °C. Sterilized seeds were first germinated on two layers of filter paper in a plastic container (10 cm × 10 cm × 5 cm) with a lid. Thirty hours after germination in darkness at 28 °C, when the roots of the seedling had reached 1–2 cm, twelve similar seedlings from each line were transferred to new containers with 30 mL of 15% *w*/*v* PEG6000/water solution or ddH_2_O. For the F_2:3_ population and the recombinants’ progenies, three replicates were performed for each line, and three replications were performed for each F_3:4_ line under PEG stress. After four days of incubation in darkness at 28 °C (five days after germination), the root length of the twelve seedlings for each line was measured with a ruler. Root samples of RA11 and RA171 after five days of PEG treatment or water treatment were collected for gene expression analysis.

### 4.4. Statistical Analysis

An analysis of variance (ANOVA) and multiple comparisons (Fisher’s unprotected LSD) were carried out based on a general linear model to test for phenotypic differences between the different genotypes. Statistical analyses were performed, and graphs were drawn using GraphPad Prism 9 (GraphPad Software, LLC, Boston, MA, USA).

### 4.5. RNA Extraction and Quantitative PCR with Reverse Transcription

Total RNA was isolated using a FastPure Universal Plant Total RNA Isolation Kit (RC411, Vazyme, Nanjing, China) according to the manufacturer’s instructions. cDNA was synthesized using a HiScript III All-in-one RT SuperMix Perfect kit for qPCR (RC333, Vazyme, Nanjing, China). Quantitative real-time PCR (qRT-PCR) was conducted on a Roche Lightcycler^®^ 96 (Roche Molecular Systems, Mannheim, Germany) instrument using ChamQ Universal SYBR qPCR Master Mix (Q711, Vazyme). The qRT-PCR volume was 20 μL, consisting of 10 μL of 2×ChamQ Universal SYBR qPCR Master Mix, 0.4 μL of each primer (10 μM), 2 μL of cDNA, and 7.2 μL of ddH_2_O. The amplification conditions were as follows: 95 °C for 30 s, followed by 40 cycles of 95 °C for 10 s and 60 °C for 30 s. Three biological replicates of each sample were tested for all tested genes. The raw qPCR data was processed using LightCycler^®^ 96 software v1.1 (Roche Molecular Systems, Mannheim, Germany). The relative gene expression levels were calculated using the 2^-∆∆CT^ method using *SiUBQ6* as an internal control [62]. The primer pairs used for quantitative PCR experiments are listed in Table S4.

## 5. Conclusions

In this study, we fine mapped the novel QTL *qRLP12* for root length under drought stress to a physical distance of 91 kb in sesame, establishing it as a key locus for root length enhancement under drought conditions. Among the annotated genes within the target region, *LOC105165547* encoding a callose synthase 7 protein is the most likely candidate gene of *qRLP12* based on their putative functions and sequence variations between two parents and expression analysis. To our knowledge, this *qRLP12* locus is the first report to refine a QTL associated with root length under drought stress in sesame. Our study paves the way for future investigations into the molecular mechanisms governing root growth under drought stress in sesame, while the linked markers developed herein can facilitate marker-assisted selection for root improvement under water deficits in sesame breeding efforts.

## Figures and Tables

**Table 1:** Statistical analysis for root length under PEG stress (RLP) of F_2:3_ population.

Genotype ^1^	No. ^2^	RLP ^3^ (cm)
JJ	23	3.80 ± 0.68 a
ZJ	56	5.26 ± 1.05 b
ZZ	27	6.05 ± 0.87 c

^1^ Genotypes identified using flanking markers *Z9* and *Z10*; ZZ, JJ and ZJ represent ZSB type, JHM type and heterozygous type. ^2^ Number of lines. ^3^ Data for RLP are shown as mean ± standard deviation. Letters represent significant differences at *p* = 0.01.

**Table 2:** Predicted candidate genes in the 91 kb target region of *qRLP12*.

Gene ID ^1^	Annotation	Variation ^2^
*LOC105165541*	subtilisin-like protease SBT5.3	NA
*LOC10516554* *2*	heavy metal-associated isoprenylated plant protein 16-like	NA
*LOC10516554* *3*	3-hydroxyisobutyryl-CoA hydrolase-like protein 5	NA
*LOC10516554* *4*	protein VACUOLELESS1	NA
*LOC10516554* *5*	GDSL esterase/lipase	NA
*LOC10516554* *6*	conserved oligomeric Golgi complex subunit 5	NA
*LOC10516554* *7*	callose synthase 7	Exon14: A1558G (Thr520Ala); Exon42: 5679indel35bp, 5684indel37bp; Exon42: T5696C (Val1910Ala)

^1^ Gene accession numbers were retrieved from the annotation of sesame reference genome (https://www.ncbi.nlm.nih.gov/datasets/gene/GCF_000512975.1/, accessed on 5 March 2024). ^2^ Numbers represent the positions of polymorphic sites in the coding region of candidate genes between JHM and ZSB, the location of start codon was set as 1, “NA” indicates no sequence variation in the coding region of annotated genes.

## Data Availability

The data used in this study are available within the article and its accompanying Supplementary Materials.

## Supplementary Materials

Supporting information can be downloaded at https://www.mdpi.com/article/10.3390/ijms26072886/s1.

## Abbreviations

The following abbreviations are used in this manuscript:

QTL	quantitative trait locus
JHM	jinhuangma
ZSB	zhushanbai
RIL	recombinant inbred line
PEG	polyethylene glycol
RLP	root length under PEG-induced drought stress
TFs	transcription factors
qRT-PCR	quantitative real-time PCR
GWAS	genome-wide association
GELP	GDSL esterase/lipase
InDels	insertions and deletions
dCAPS	derived cleaved amplified polymorphic sequences
CAPS	cleaved amplified polymorphic sequences
CALS7	CALLOSE SYNTHASE 7
