# Supplementary figures and images for: Fine Mapping of the QTL qRLP12 That Controls Root Length Under Polyethylene glycol-Induced Drought Stress During the Early Seedling Stage of Sesame
Source: Int J Mol Sci. 2025 Mar 22;26(7):2886. doi: 10.3390/ijms26072886 (PMC11988704; doi:10.3390/ijms26072886)

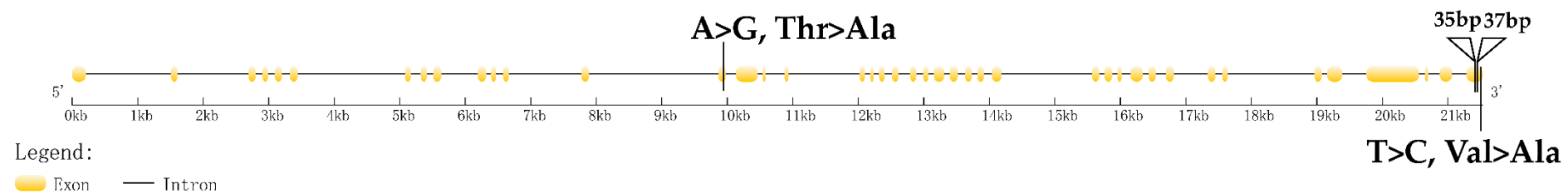

**Figure S1.** Gene structure and locations of missense mutation and two InDels in *SiCalS7* (*LOC105165547*)

Supplement: Supplementary file 1 [file ijms-26-02886-s001.zip › Suppl. Figures.pdf]
